# P07-13 Barriers and opportunities to improve collaboration between healthcare and physical activity professionals involved in prescribing physical activity for inactive people in the Netherlands

**DOI:** 10.1093/eurpub/ckac095.113

**Published:** 2022-08-29

**Authors:** Dorine Collard, Kirsten Gutter

**Affiliations:** Mulier Institute, Utrecht, The Netherlands

**Keywords:** Healthcare, Referral, Inactive, Physical activity, Professionals

## Abstract

**Background:**

Healthcare professionals play an important role in motivating inactive people with a chronic disease (e.g. diabetes, cancer, heart disease) for physical activity. They can initiate a discussion about physical activity with the patient and refer to a physical activity (PA)-professional for more detailed action-planning through ‘physical activity on prescription'. However, the collaboration between healthcare and PA-professionals to refer and guide patients to physical activities is in its infancy and can be improved. To enhance this collaboration the aim of our study was to evaluate how the collaboration is shaped and what barriers were experienced.

**Methods:**

We conducted an exploratory study in which quantitative and qualitative research methods were used. Two online questionnaires were distributed among PA-professionals in the Netherlands (spring and autumn 2021). Questionnaires were returned by respectively 209 and 116 respondents. Questions on how the collaboration was shaped, how the collaboration worked and what barriers were experienced were asked. Besides that, two focus group discussions were held with PA-professionals (n = 8) focussing on barriers and opportunities to improve ‘Physical activity prescription'.

**Results:**

More than half of the Dutch PA-professionals indicate that they collaborate with healthcare professionals in primary care (56%) to guide patients to physical activities. Far fewer PA-professionals collaborate with healthcare professionals in secondary care (22%). Half of the respondents indicate that they experience the collaboration in physical activity prescription as insufficient (47%). Most important barriers are determined, for example healthcare and PA-professionals are unfamiliar with each other; there is uncertainty about roles and tasks of professionals involved; there is a lack of communication; PA-professionals sometimes do not have the skills to guide people with a chronic disease. Finally, a lack of time and budget limits the collaboration between professionals involved in physical activity on prescription.

**Conclusions:**

The collaboration between professionals to refer and guide inactive people with a chronic disease from the healthcare setting to physical activities can be improved by responding to the barriers that are indicated by this study. This study contributes to improving health-enhancing physical activity in an inactive target group and decreasing the prevalence of physical inactivity.

